# Albumin: a mediator of the association between serum calcium and triglyceride-glucose index among Chinese individuals with osteoporotic fractures

**DOI:** 10.3389/fendo.2025.1574059

**Published:** 2025-09-12

**Authors:** Jia-qi Liang, Jian Xu, Yan Cao, Yin-lin Wei, Guo-ji Lin, Jian Jin, Chong Li, Ke Lu

**Affiliations:** ^1^ Department of Orthopedics, Affiliated Kunshan Hospital of Jiangsu University, Suzhou, Jiangsu, China; ^2^ Kunshan Biomedical Big Data Innovation Application Laboratory, Suzhou, Jiangsu, China; ^3^ Department of Orthopedics, The First People’s Hospital of Kunshan, Gusu School, Nanjing Medical University, Suzhou, Jiangsu, China; ^4^ Department of Nuclear Medicine, Nanjing First Hospital, Nanjing Medical University, Nanjing, Jiangsu, China; ^5^ Kunshan Municipal Health and Family Planning Information Center, Suzhou, Jiangsu, China

**Keywords:** osteoporotic fractures, serum Ca, TyG index, albumin, mediator

## Abstract

**Background:**

Osteoporotic fractures (OPFs) are a major global health concern, affecting nearly 200 million individuals worldwide. Notably, patients with type 2 diabetes mellitus (T2DM) face a heightened fracture risk, even when bone mineral density (BMD) is normal or elevated. This study investigated the associations between serum calcium (Ca), triglyceride-glucose (TyG) index, albumin, and their interactions in Chinese patients with OPFs.

**Methods:**

This cross-sectional study included 1,541 participants who were 50 years of age or older, recruited from the Affiliated Kunshan Hospital of Jiangsu University between January 2017 and August 2023. The primary independent and dependent variables were serum Ca and the TyG index, respectively. Albumin served as the mediating factor in this analysis. The relationship between serum Ca or albumin and the TyG index was investigated using multivariable logistic regression analyses. A mediation analysis was conducted to ascertain whether albumin mediated the relationship between serum Ca and the TyG index.

**Results:**

The study revealed a positive correlation between serum Ca and TyG index, with each unit increase in serum Ca associated with a 0.903 increase in TyG index. Albumin partially mediated this relationship, accounting for approximately 21.74% of the effect of serum Ca on TyG index. The models demonstrated a consistent association across various adjustments for confounding variables.

**Conclusions:**

Findings suggest a mediation link between serum Ca and albumin and the risk of TyG index. The significance of albumin as a mediator deserves recognition and consideration.

## 1 Introduction

Osteoporotic fractures (OPFs) pose a significant global health challenge (1). Disruption of the equilibrium between osteoblast-mediated bone formation and osteoclast-driven bone resorption leads to structural bone degradation and increased fracture susceptibility. Osteoporosis affects approximately 200 million individuals worldwide (2), with prevalence rates of 6.46% in Chinese men and 29.13% in women aged ≥ 50 years (3). Furthermore, a recent review highlighted that 37.8% of individuals with diabetes in China are affected by osteoporosis (4). Notably, despite normal or even elevated bone mineral density (BMD) in many patients with type 2 diabetes mellitus (T2DM) (5), their fracture risk surpasses that of non-diabetic individuals (6, 7). These findings underscore the urgent need to identify modifiable factors related to glycometabolic indices in patients with OPFs.

Serum calcium (Ca) is crucial for human health (8), and its role in the regulation of glucose homeostasis has been well-established (9). Disruptions in serum Ca balance have been linked to insulin dysfunction and glucose regulation abnormalities, potentially contributing to the onset of type 2 diabetes mellitus (T2DM) (10–12). The triglyceride-glucose (TyG) index is a recognized marker for estimating insulin levels (13, 14) and is considered more reliable than the homeostasis model assessment of insulin resistance in healthy individuals (15). Elevated levels of the TyG index have been associated with an increased risk of metabolic disorders (16), which can significantly influence bone health (17). Recent studies have suggested that heightened insulin resistance, reflected by a high TyG index, may adversely affect bone quality and contribute to the pathogenesis of osteoporosis (18, 19). Several epidemiological studies have reported a correlation between serum Ca and TyG index (20, 21). However, the potential mediating role of albumin in this association has not been well established in the existing literature. To the best of our knowledge, there are currently limited studies exploring whether albumin acts as a mediator between serum Ca and TyG index. Therefore, our study was designed to further investigate the relationship between serum Ca and the TyG index and to explore the possible mediating effect of albumin in this association.

Albumin is a crucial protein in serum, responsible for maintaining oncotic pressure and facilitating the transport of various substances throughout the body. Its role in regulating oncotic pressure is particularly significant, as albumin accounts for approximately 75% of plasma colloid osmotic pressure, which is essential for fluid balance and the distribution of body fluids across compartments (22, 23). A recent study also showed that TyG index is associated with a significant increase in albumin (24). This correlation suggests that albumin may act as a mediator in the metabolic pathways linking triglyceride and glucose metabolism. For instance, studies have indicated that elevated levels of the TyG index correspond with increased albumin concentrations, thereby supporting the hypothesis that albumin mediates pathways linking triglyceride and glucose metabolism (25, 26). However, no research has yet explored whether the relationship between serum Ca and TyG index is mediated by albumin.

In this study, we hypothesize that serum Ca are significantly associated with the TyG index in patients with OPFs, and that albumin may mediate this relationship. Furthermore, we specifically aim to address the following research questions: (1) What is the nature of the relationship between serum Ca and the TyG index in this patient population? (2) Does albumin serve as a mediating factor in this association? By exploring these associations, we hope to identify potential metabolic targets for intervention, which could lead to more effective prevention strategies for OPFs, thereby reducing the overall burden of this disease on individuals and the healthcare systems. This cross-sectional study will explore the metabolic components influencing bone health and fracture risk among the elderly population in China.

## 2 Materials and methods

### 2.1 Populations

This study employed an open enrollment design and was conducted as a retrospective real-world investigation at the Affiliated Kunshan Hospital of Jiangsu University (AKHJU). AKHJU is the largest tertiary-level A hospital in the Kunshan area, which serves over 3,000,000 inhabitants of Kunshan City, Jiangsu Province, China. The study participants were identified from the Regional Health Registration Platform (RHRP) of Kunshan City and the Population Death Registration System (PDRS) of Jiangsu Province. We obtained electronic patient records from all patients ≥50 years of age who had recently been diagnosed with an osteoporosis fracture (fracture of the wrist, proximal humerus, hip, or vertebra) requiring hospitalization between January 1, 2017, and August 21, 2023. In our study, we utilized a comprehensive database from a previous Fracture Liaison Service (FLS) study, which included 4,782 OPFs patients (27). The Fracture Liaison Service of Kunshan (FLS-KS) Model is a cloud-based chronic disease management system designed to improve osteoporosis care. The system consists of three modules: (1) a management and statistical platform integrated with hospital information and laboratory systems for automatic patient data capture, generation of individualized treatment plans, and follow-up schedules; (2) a mobile workstation (via the WeCom app) for medical staff to monitor patient status, ensure timely evaluation, and respond to adverse events; and (3) a patient mobile client (via WeChat) providing educational resources, treatment information, follow-up scheduling, a medication adherence punch-in module with incentives, and a channel for patients to report adverse events. All patients had been hospitalized and underwent surgical intervention. OPFs, or fragility fractures, are fractures resulting from low-impact events, such as falling from a standing position or from a relatively low height. They can significantly elevate the likelihood of additional fractures because of their enhanced vulnerability (28). For the diagnosis of OPFs, it is essential to have osteoporosis and rule out other metabolic bone disorders (29). Osteoporosis was identified based on the following conditions: (1) presence of bone weakness and fractures without other metabolic bone conditions, along with a standard BMD (T-score); (2) confirmation of osteoporosis with a T-score of −2.5, even if no current bone fractures were present (30).

**Figure 1:**
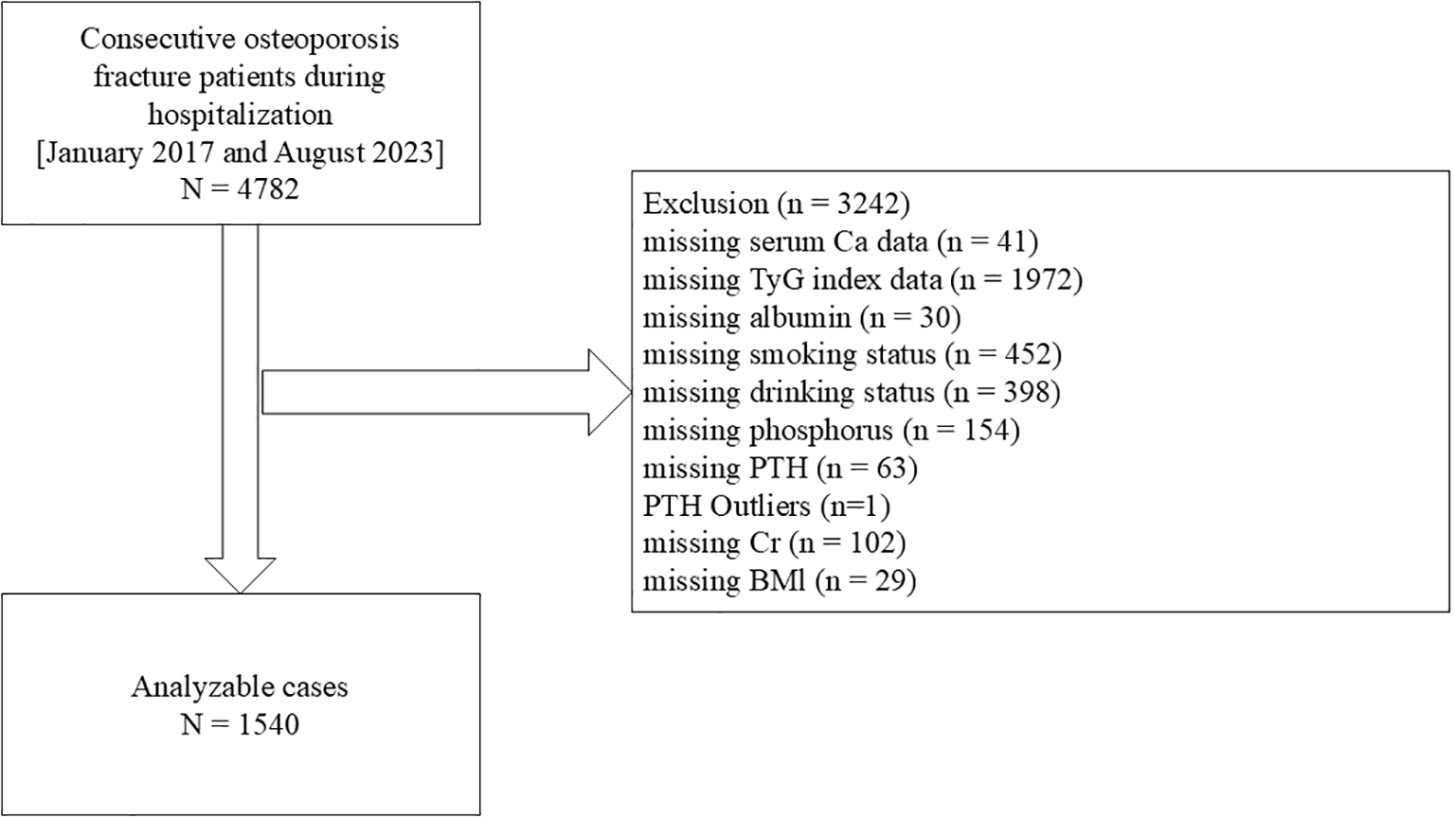
Flowchart of participants selection. Ca, calcium; TyG, triglyceride-glucose; BMI, body mass index; PTH, parathyroid hormone; Cr, creatinine.

Our study group consisted exclusively of adults aged ≥ 50 years (n = 4,782). We excluded patients with missing serum Ca data (n = 41) and incomplete TyG index information (n = 1,972). Furthermore, individuals without covariate data (n = 1,228) were also removed (31). Ultimately, 1,541 participants were assessed and included in the study (**Figure 1**). The ethical approval number for this study is 2024 - 03-053-H00-K01, and it strictly adhered to the Declaration of Helsinki. To maintain objectivity, all patient information was anonymized. Written authorization with full disclosure was obtained from each patient.

### 2.2 Serum Ca

Serum Ca was quantitatively determined using the Beckman Coulter AU5800 clinical chemistry analyzer. The arsenazo III method was used to measure serum Ca. This colorimetric technique is based on the reaction between serum Ca ions in the serum and the Arsenazo III reagent, which results in a complex that absorbs light at a specific wavelength. The color intensity generated was directly correlated with the serum Ca concentration.

### 2.3 TyG index assessment

The formula for calculating the TyG index is Ln [fasting triglyceride (mg/dL) × fasting plasma glucose (FPG; mg/dL)/2] (32). Blood samples collected after overnight fasting were analyzed using a Beckman AU5800 biochemical analyzer. Triglyceride levels were measured using the GPO-POD method, while fasting plasma glucose levels were assessed using a hexokinase-based reaction.

### 2.4 Albumin assessment

Albumin was measured using the Beckman AU5800 biochemical analyzer, employing the Biuret method for detection.

### 2.5 Covariates

This study identified covariates guided by clinical expertise and evidence from previous research. Accordingly, the variables considered included age, gender, BMI, hypertension, diabetes, smoking status and drinking status and levels of phosphorus, parathyroid hormone (PTH), and creatinine (Cr) (20). Age and gender were obtained from medical records. Smoking was defined as being a current or former smoker within the last 12 months. Drinking was defined as drinking once per week for the previous 12 months. BMI was computed as the weight in kilograms divided by the square of the height in meters. Phosphorus was measured using the Beckman AU5800 biochemical analyzer by the phosphomolybdate method. PTH was measured using the Beckman DXI800 chemiluminescence analyzer by the chemiluminescence method. Cr was measured using the fully automated coagulation analyzer CN - 6000 by the sarcosine oxidase method. Serum phosphorus levels were measured using standard laboratory protocols.

All blood specimens were obtained from fasting patients who had fasted. All assessments were conducted using the initial fasting blood samples obtained within 24 hours of hospital admission.

### 2.6 Statistical analysis

Continuous variables and categorical data are presented as mean ± SD, medians (25th, 75th), and count (%), respectively. Univariate analyses of single-variable data in absolute values were conducted using Fisher’s exact test or the chi-square test. T-tests were applied to continuous variables with normal distributions, whereas the Mann-Whitney U test was used for non-normally distributed continuous data. Univariate linear regression analyses were performed on serum Ca and TyG index in patients with OPF_S_ to investigate their direct relationship. This was accomplished using generalized estimating equations (GEE) with suitable modifications for confounding variables. The generated models underwent full (Model 3), partial (Model 2), and no adjustment (Model 1). Initially, collinearity diagnosis was conducted using the variance inflation factor (VIF). The need for covariate adjustment was then assessed according to the following criteria: Criterion 1 involved introducing covariates to the basic model, which initially comprised only diabetes status and trochanteric BMD, or removing covariates from the full mode. The full model incorporated all potential covariates including age, gender, BMI, magnesium, sodium, phosphorus, platelet count, hemoglobin, albumin, Ca, neutrophil count, lymphocyte count, monocyte count, alanine aminotransferase (ALT), aspartate aminotransferase (AST), Cr, blood urea nitrogen (BUN), serum uric acid (SUA),PTH, hypertension, diabetes, American Society of Anesthesiologists (ASA) scores and Charlson comorbidity index (CCI), and fracture category, in addition to diabetes status and trochanteric BMD. A covariate was considered necessary for adjustment if its inclusion or exclusion resulted in a ≥10% change in the odds ratio (OR). Criterion 2 required meeting Criterion 1 or having a covariate with a *P*-value < 0.1 in the univariate model (33). Model 1 with no adjustments, Model 2 with modifications for age, gender, and BMI, While Model 3 was adjusted for age, gender, BMI, phosphorus, Cr, PTH, hypertension, diabetes, smoking status and drinking status. A generalized additive model (GAM) was employed to detect potential non-linear associations. Significant associations prompted the use of a segmented linear regression model to explore threshold effects in the smoothed curves. Additional analyses were performed to verify the robustness of the findings and examine the differences between patient groups based on specific confounding factors. Modified tests were conducted using likelihood ratio tests (LRT).

A mediation analysis explored whether albumin mediated the relationship between serum Ca (treatment variable) and TyG index (outcome variable). This analysis quantified the total effect (association between serum Ca and TyG index), natural direct effect (the effect of serum Ca on TyG index excluding albumin), and natural indirect effect (the influence of serum Ca on TyG index via albumin). The mediation analysis was adjusted for covariates, including age, gender, BMI, phosphorus, Cr, PTH, hypertension, diabetes, smoking status and drinking status, using three distinct models. A *P*-value < 0.05 was considered statistically significant.

Empower Stats (X&Y Solutions, Inc., Boston, MA, USA) was utilized for all statistical evaluations. Furthermore, R 3.6.3 was employed. P-values were deemed statistically significant if they were below 0.05.

## 3 Results

### 3.1 Patient attributes

**Table 1** outlines the fundamental attributes of 1540 OPF_S_ patients admitted from January 2017 to August 2023 and categorized into specific quartiles. Among the participants, 25.52% were men and 74.48% were women, with a median age was 68 years, with the 25th percentile at 61 and the 75th percentile at 78 years. The median serum calcium level was 2.21 mmol/L, with the 25th and 75th percentiles at 2.13 mmol/L and 2.30 mmol/L, respectively. Patients were categorized into four quartiles according to serum Ca (1.47 - 2.12, 2.13 - 2.20, 2.21 - 2.29, 2.30 - 2.91).

**Table 1:** Patient characteristics based on serum calcium quartiles.

Characteristics	Median (25th, 75th)	Mean + SD/N (%)				*P*-value
		Serum calcium 4 quantiles				
	Total	Q1 (1.47 - 2.12)	Q2 (2.13 - 2.20)	Q3 (2.21 - 2.29)	Q4 (2.30 - 2.91)	
N	1540	377	386	387	390	
Age, years	68.00 (61.00, 78.00)	68.94 ± 11.21	68.56 ± 10.79	69.88 ± 11.35	70.30 ± 10.20	0.096
Gender, N						0.689
Female	1147 (74.48%)	277 (73.47%)	282 (73.06%)	290 (74.94%)	298 (76.41%)	
Male	393 (25.52%)	100 (26.53%)	104 (26.94%)	97 (25.06%)	92 (23.59%)	
BMI, kg/m^2^	23.12 (20.94, 25.36)	23.37 ± 3.27	23.25 ± 3.30	23.17 ± 3.27	23.26 ± 3.29	0.869
Smoking status, N						0.575
No	1459 (94.74%)	360 (95.49%)	369 (95.60%)	363 (93.80%)	367 (94.10%)	
Yes	81 (5.26%)	17 (4.51%)	17 (4.40%)	24 (6.20%)	23 (5.90%)	
Drinking status, N						0.281
No	1495 (97.08%)	361 (95.76%)	377 (97.67%)	379 (97.93%)	378 (96.92%)	
Yes	45 (2.92%)	16 (4.24%)	9 (2.33%)	8 (2.07%)	12 (3.08%)	
Hypertension N						0.679
No	1311 (85.13%)	320 (84.88%)	322 (83.42%)	334 (86.30%)	335 (85.90%)	
Yes	229 (14.87%)	57 (15.12%)	64 (16.58%)	53 (13.70%)	55 (14.10%)	
Diabetes N						0.374
No	1467 (95.26%)	357 (94.69%)	365 (94.56%)	375 (96.90%)	370 (94.87%)	
Yes	73 (4.74%)	20 (5.31%)	21 (5.44%)	12 (3.10%)	20 (5.13%)	
PTH, ng/L	12.21 (9.46, 16.26)	15.31 ± 11.14	14.04 ± 7.77	13.90 ± 8.47	14.37 ± 9.18	0.192
Cr, μmol/L	60.00 (51.00, 73.00)	68.29 ± 39.14	63.65 ± 20.53	65.19 ± 45.72	62.65 ± 18.22	0.101
Serum calcium, mmol/L	2.21 (2.13, 2.30)	2.05 ± 0.08	2.17 ± 0.02	2.25 ± 0.03	2.37 ± 0.08	<0.001
Phosphorus, mmol/L	1.06 (0.93, 1.18)	1.00 ± 0.20	1.04 ± 0.19	1.07 ± 0.20	1.10 ± 0.22	<0.001
Albumin, g/L	40.10 (37.68, 42.50)	37.11 ± 3.85	39.19 ± 3.16	40.69 ± 3.05	42.79 ± 3.37	<0.001

TyG, triglyceride-glucose; 25th, 25th percentile; 75th, 75th percentile; SD, standard deviation; Q1, first quartile; Q2, second quartile; Q3, third quartile; Q4, fourth quartile; BMI, body mass index; PTH, parathyroid hormone; Cr, creatinine.

*P*-value*: Kruskal Wallis Rank Test for continuous variables, Fisher Exact for categorical variables with Expects < 10.

### 3.2 Univariate analyses of factors associated with TyG index

Univariate analysis revealed notable associations between TyG index and factors such as age, gender, BMI, phosphorus, Cr, PTH, hypertension, diabetes, smoking status and drinking status (**Table 2**).

**Table 2:** Univariate analyses of factors associated with TyG index.

Characteristics	TyG index	
	β^a^ (95% CI)	*P*-value
Age, years	-0.001 (-0.004, 0.002)	0.628
Gender, N		
Female	Reference	
Male	0.017 (-0.053, 0.088)	0.630
BMI, kg/m^2^	-0.012 (-0.022, -0.003)	0.009
Smoking status		
No	Reference	
Yes	0.035 (-0.103, 0.172)	0.623
Drinking status		
No	Reference	
Yes	0.053 (-0.129, 0.236)	0.567
Hypertension		
No	Reference	
Yes	0.017 (-0.069, 0.104)	0.695
Diabetes		
No	Reference	
Yes	0.103 (-0.041, 0.247)	0.163
PTH, ng/L	0.001 (-0.003, 0.003)	0.987
Cr, μmol/L	0.001 (0.000, 0.002)	0.011
Serum calcium, mmol/L	0.873 (0.645, 1.101)	<0.001
Serum calcium 4 quantiles, mmol/L		
Q1 (0.74 - 2.11)	Reference	
Q2 (2.12 - 2.19)	0.143 (0.057, 0.229)	0.001
Q3 (2.20 - 2.28)	0.163 (0.077, 0.249)	<0.001
Q4 (2.29 - 2.91)	0.306 (0.220, 0.391)	<0.001
Phosphorus, mmol/L	0.112 (-0.039, 0.263)	0.145
Albumin, g/L	0.023 (0.015, 0.030)	<0.001

a Dependent variable TyG index, as a result of univariate analyses for TyG index. TyG, triglyceride-glucose; BMI, body mass index; PTH, parathyroid hormone; Cr, creatinine.

### 3.3 Exploring the association between serum Ca and TyG index

Multiple linear regression analyses were conducted, and the results of the three different models are presented in **Table 3**. All models revealed a significant positive relationship between serum Ca and TyG index (*P*-value < 0.001). After adjusting for potential confounders, each one-unit increase in serum Ca was associated with a 0.903 increase in TyG index. This association remained statistically significant (*P*-value for trend < 0.001) when the serum Ca was divided into quartiles.

**Table 3:** Association between serum calcium and TyG index in different models.

	Model 1^a^ β (95% CI) *P*-value	Model 2^b^ β (95% CI) *P*-value	Model 3^c^ β (95% CI) *P*-value
TyG index			
serum calcium, mmol/L	0.873 (0.645, 1.101) <0.001	0.876 (0.648, 1.104) <0.001	0.903 (0.671, 1.135) <0.001
serum calcium 4 quantiles, mmol/L			
Q1 (1.47-2.12)	Reference	Reference	Reference
Q2 (2.13-2.20)	0.143 (0.057, 0.229) 0.001	0.141 (0.055, 0.227) <0.001	0.147 (0.061, 0.233) <0.001
Q3 (2.21-2.29)	0.163 (0.077, 0.249) <0.001	0.163 (0.080, 0.249) <0.001	0.171 (0.084, 0.257) <0.001
Q4 (2.30-2.91)	0.306 (0.220, 0.391) <0.001	0.307 (0.221, 0.393) <0.001	0.316 (0.229, 0.404) <0.001
* P* for trend	<0.001	<0.001	<0.001

Association between serum calcium and TyG index in different models. ^a^No adjustment. ^b^Adjusted for age, gender, BMI. ^c^Adjusted for age, gender, BMI, phosphorus, Cr, PTH, hypertension, diabetes, smoking status and drinking status. TyG, triglyceride-glucose; BMI, body mass index; Cr, creatinine; PTH, parathyroid hormone.

### 3.4 Spline smoothing plot and threshold assessments

**Figure 2:**
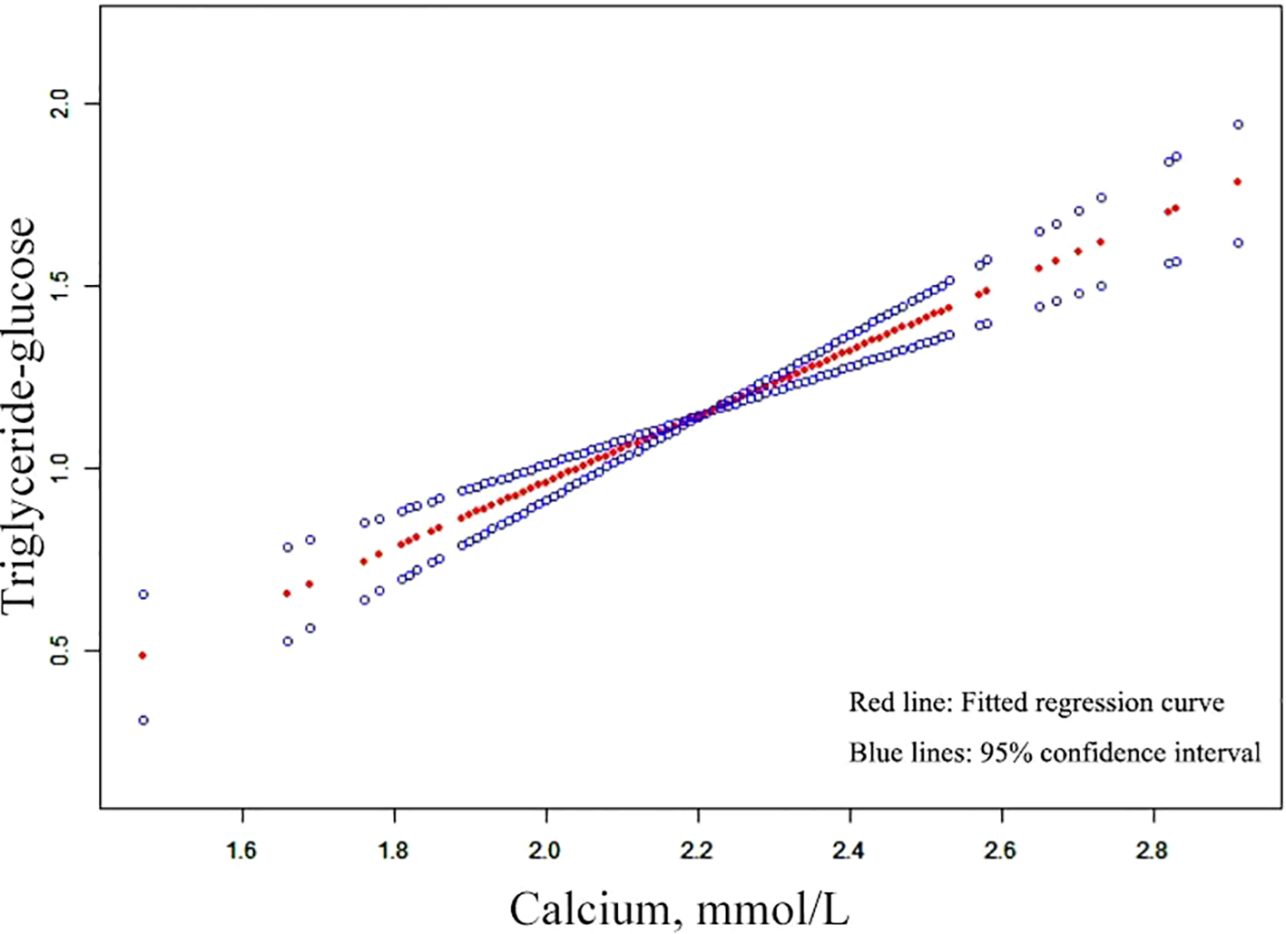
Curves adjusted for smoothing that illustrate the association between serum Ca and TyG index. The upper and lower curves indicate the boundaries of the 95% CI, while the central curve depicts the relationship between the serum Ca and TyG index. Models were adjusted for a age, gender, BMI, phosphorus, Cr, PTH, hypertension, diabetes, smoking status and drinking status. Ca, calcium; TyG, triglyceride-glucose; BMI, body mass index; PTH, parathyroid hormone; Cr, creatinine.

The relationship between serum Ca and TyG index was assessed in 1540 individuals using a graphical representation (**Figure 2**). Threshold assessment was further conducted after adjusting for age, gender, BMI, phosphorus, Cr, PTH, hypertension, diabetes, smoking status and drinking status (**Supplementary Table S1**). Nonetheless, a direct link was identified between serum Ca and TyG index. The final model, accounting for age, gender, BMI, phosphorus, Cr, PTH, hypertension, diabetes, smoking status and drinking status, revealed an effect magnitude of 0.907 (95% CI: 0.675 - 1.139, *P*-value < 0.001). Additionally, threshold assessment based on curve fitting did not show any confirmed inflection point for non-linear association (**Supplementary Table S1**) (*P*-value > 0.05).

### 3.5 Association between serum Ca and albumin

**Supplementary Table S2** showed the association of serum Ca with albumin in the entire cohort. We used a linear regression model to evaluate the associations between serum Ca and albumin. In the fully adjusted model, serum Ca showed a positive association with albumin (*P*-value < 0.001).

### 3.6 Association albumin and TyG index

We also constructed three logistic regression models to explore the relationship between albumin and TyG index. All models exhibited a positive correlation between albumin and TyG index (*P*-value < 0.001) (**Supplementary Table S3**).

### 3.7 Subgroup analysis

This study stratified subgroups by gender and FLS to further validate the reliability of the resultant outcomes in the fully adjusted model when potential confounding variables were represented. Adjustment was established for those covariates that were not utilized for stratification. The studies showed consistent patterns in the results, with no detected interactions due to stratification (all *P*-value < 0.001, **Supplementary Table S4**).

### 3.8 Path analysis

**Figure 3:**
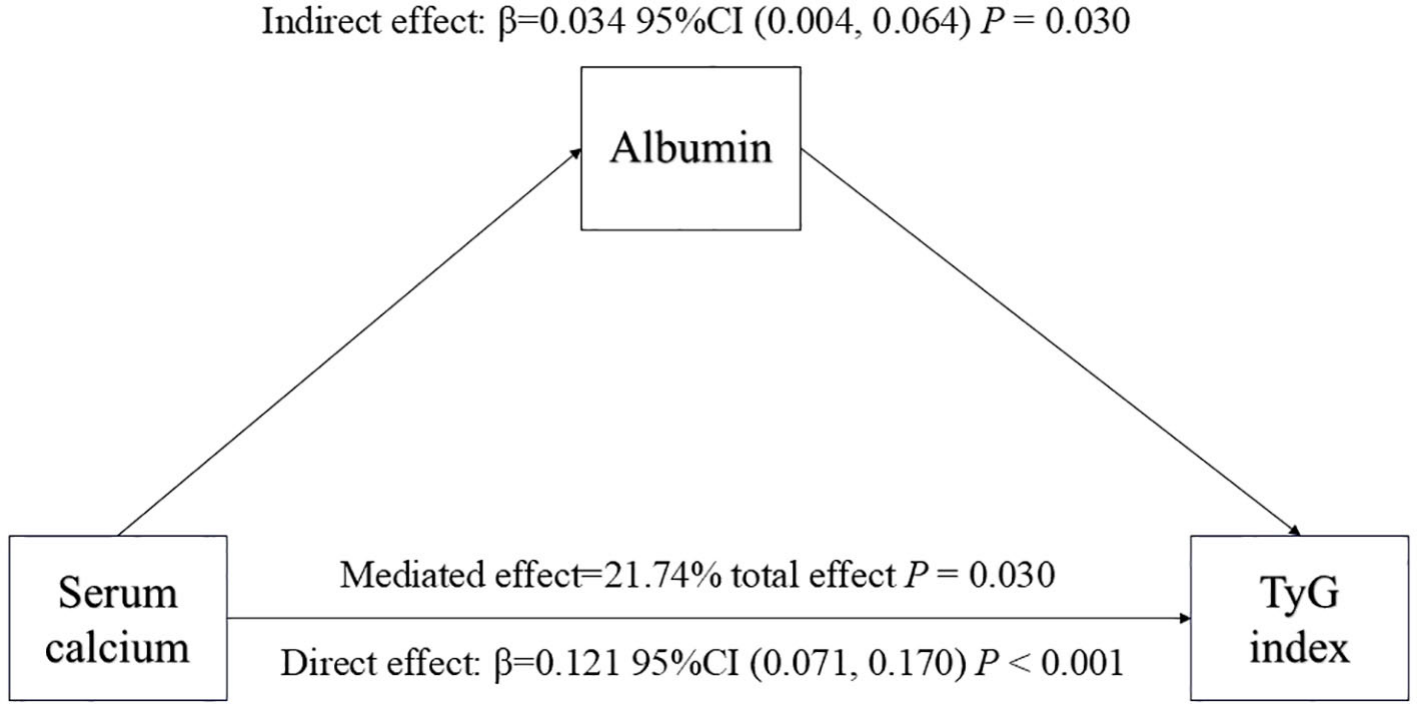
Mediation of albumin on the association between serum calcium and TyG index. **Figure 3** indicated that serum calcium had a significant direct effect on TyG index (β=0.121, 95% CI: 0.071, 0.170), and albumin partly mediated the indirect effect of serum calcium on TyG index (β=0.034, 95% CI: 0.004, 0.064). Therefore, approximately 21.74% of the serum calcium effect on TyG index was mediated through albumin levels. Zero was not included in 95% confidence intervals representing statistical significance.

As shown in **Table 4** and **Figure 3**, our findings indicate that serum Ca had a significant direct impact on TyG index (95% CI: 0.071 - 0.170, *P*-value < 0.001). Furthermore, albumin was identified as a partial mediator of the effect of serum Ca on TyG index (95% CI: 0.004 - 0.04, *P*-value = 0.030). Specifically, approximately 21.74% of the impact of serum Ca on TyG index was mediated by albumin. These results hold true after adjusting for various potential confounders including age, gender, BMI, phosphorus, Cr, PTH, hypertension, diabetes, smoking status and drinking status. The findings remained consistent even when only demographic variables were included.

**Table 4:** Mediation analysis of the association between serum calcium and TyG index mediated by albumin.

Exposure	Model 1^a^ β (95% CI) *P*-value	Model 2^b^ β (95% CI) *P*-value	Model 3^c^ β (95% CI) *P*-value
Direct effect	0.122 (0.074, 0.171) <0.001	0.122 (0.074, 0.171) <0.001	0.121 (0.071, 0.170) <0.001
Indirect effect	0.026 (-0.005, 0.058) 0.096	0.026 (-0.005, 0.058) 0.096	0.034 (0.004, 0.064) 0.030
Total effect	0.148 (0.110, 0.191) <0.001	0.148 (0.110, 0.191) <0.001	0.154 (0.116, 0.197) <0.001
PM, %	17.68	17.68	21.74
*P*-value	0.096	0.096	0.030

a No adjustment.

b Adjusted for age, gender, BMI.

c Adjusted for age, gender, BMI, phosphorus, Cr, PTH, hypertension, diabetes, smoking status and drinking status. TyG, triglyceride-glucose; BMI, body mass index; Cr, creatinine; PTH, parathyroid hormone.

## 4 Discussion

We aimed to examine the relationships between TyG index, serum Ca, and albumin, as well as to evaluate whether albumin was associated with both serum calcium levels and the TyG index. Our analysis revealed positive correlations between serum Ca and TyG index. Mediation analysis demonstrated that serum Ca had a direct influence on TyG index, and albumin partially mediated the effect of serum Ca on TyG index. Approximately 21.74% of the effect of serum Ca effect on TyG index was mediated by albumin. Interventions aimed at optimizing serum Ca and albumin might not only help in reducing the risk of developing insulin resistance, which is prevalent in this demographic, but also in strengthening the skeletal system. This could potentially lead to the development of tailored nutritional and pharmacological strategies that address both metabolic dysfunction and bone health in OPFs patients.

Osteoporosis is an increasingly significant public health issue (34–36). A recent cross-sectional study in the Chinese population revealed osteoporosis prevalence rates of 5.0% and 20.6% among males and females aged 40 years and older, respectively. Osteoporosis notably elevates the financial strain of individuals experiencing severe fractures and chronic pain (37). OPFs are prevalent contributors to illness and death among elderly individuals (38–40).

Our study elucidates the complex interactions between serum Ca, TyG index, and albumin, revealing a mechanistically interconnected pathway that aligns with existing literature (10–12, 35, 36). Numerous studies have established a positive association between serum Ca and TyG index, supported by the role of serum Ca in insulin secretion and its essential function in insulin-mediated intracellular processes in muscle and fat tissues (30–32). Insulin secretion is fundamentally a Ca-dependent process, with a precise intracellular serum Ca concentration required for optimal insulin function. Variations in this concentration can lead to peripheral insulin resistance by disrupting the insulin signaling cascade and reducing glucose transporter activity (33, 34). Furthermore, our findings corroborate previous research indicating that serum Ca can bind to albumin, which is positively associated with TyG index (36). This suggests that albumin not only plays a role in the metabolic processing of lipids and glucose but also functions as a mediator in the link between serum Ca and TyG index (35). Although clinical trials have not proven albumin to be a mediator in the link between serum Ca and TyG index, our findings suggest otherwise. Based on the data in **Table 4**, the proportion of the association between serum calcium and the TyG index that was potentially attributable to albumin was 21.74%, indicating that part of the effect of serum Ca on TyG index may be mediated through albumin. This could be explained by metabolic regulation involving both serum Ca and albumin.

Protein-bound serum Ca makes up 39.5% of serum Ca (41) and is the serum Ca that binds to plasma proteins (mainly albumin) (42). However, the mechanisms underlying the association between serum albumin and metabolic abnormalities remain unclear. However, one possible explanation is dietary protein intake. Increased protein consumption is positively correlated with serum albumin (43, 44), and a high-protein diet triggers the release of glucagon and insulin, accelerates glycogen turnover, and enhances gluconeogenesis (45, 46). As a result, protein-rich diets are associated with a higher risk of diabetes (47). Overall, excessive protein intake may contribute to the development of metabolic syndrome.

Understanding the relationship between serum Ca and the TyG index extends our comprehension of metabolic risk factors in patients with OPFs. The identification of albumin as a significant mediator in this association is a novel and important finding in the field. Traditionally, metabolic risk management in OPFs patients has focused on glucose and lipid profiles or bone-specific parameters. Our results suggest that the interplay between calcium metabolism and metabolic dysfunction involves albumin as a mechanistic link, thereby broadening the perspective on risk assessment and intervention.

The clinical implications of these findings are considerable. Since albumin is routinely measured and, unlike many metabolic markers, is modifiable through interventions aimed at improving nutritional status and reducing chronic inflammation, targeting albumin could become an accessible and practical strategy for mitigating metabolic risk in OPFs patients. This may complement conventional therapies focused on bone density and fracture prevention, paving the way for more integrative and personalized management approaches. Moreover, regular monitoring of both albumin and serum Ca metabolism may enable earlier identification of patients at highest risk for metabolic complications, thus informing proactive clinical interventions.

Our investigation into the association between serum Ca and albumin and TyG index showed that albumin mediates 21.74% of the influence of serum Ca on TyG index. These findings are clinically significant in two ways. First, they provide insights into the specific mechanisms through which serum Ca affect TyG index. Second, they offer a novel clinical strategy for the prevention and management of insulin resistance. By regulating serum Ca and albumin, insulin resistance may be alleviated. However, it is essential to recognize that other unidentified factors may account for the remaining 74.80% of the relationship between serum Ca and TyG index.

While our findings provide valuable insights, they have the inherent limitations of observational studies, including potential confounders and the inability to establish causality. Future studies should examine these associations using longitudinal and interventional approaches to determine the causal roles of serum Ca and albumin in TyG index and their effects on clinical outcomes in individuals with osteoporosis.

Our study’s main strengths include a large sample size, being the first to investigate albumin as a mediator between serum calcium and TyG index, and the use of multiple statistical methods to analyze their interactions. However, as a cross-sectional study, we cannot establish causality. Selection bias may exist due to our focus on participants with OPFs, and medication use could have affected outcomes despite our adjustments. Additionally, we did not assess the impact of chronic diseases such as diabetes or hypertension on the TyG index. Furthermore, while GEE account for within-subject correlations, they do not control for unmeasured confounding. Similarly, our mediation analysis, being based on cross-sectional data, cannot establish causality or the direction of effects, and is susceptible to unmeasured confounding, particularly of the mediator-outcome relationship. These limitations highlight the need for cautious interpretation of our findings and further investigation using longitudinal or experimental designs.

## 5 Conclusion

Serum Ca had a positive relationship with TyG index, and albumin played a partial role in mediating this connection in OPFs patients. This finding provides a new way to prevent the occurrence and progression of high TyG index clinically. Clinically, controlling serum Ca may help prevent or reduce a high TyG index, whereas maintaining appropriate serum albumin could further diminish the associated risk.

## Data Availability

The original contributions presented in the study are included in the article/**Supplementary Material**. Further inquiries can be directed to the corresponding author.
