# Supplementary material for: Albumin: a mediator of the association between serum calcium and triglyceride-glucose index among Chinese individuals with osteoporotic fractures
Source: Front Endocrinol (Lausanne). 2025 Sep 12;16:1574059. doi: 10.3389/fendo.2025.1574059 (PMC12463993; doi:10.3389/fendo.2025.1574059)
Supplement: Supplementary file 1 [file Table1.docx]

**Table S1.** Threshold analyses examining the relationship between serum calcium and TyG index

|  | Model 3^a^ |
| --- | --- |
|  | β (95% CI) *P*-value |
| Model A^b^ |  |
| One line slope | 0.907 (0.675, 1.139) <0.001 |
| Model B^c^ |  |
| TyG index turning point (K) | 2.41 |
| ＜K | 0.972 (0.710, 1.234) <0.001 |
| ＞K | 0.329 (-0.780, 1.438) 0.561 |
| Slope 2–Slope 1 | -0.643 (-1.849, 0.564) 0.297 |
| TyG value at K | 1.338 (1.277, 1.400) |
| LRT test^d^ | 0.295 |

^a^ Adjusted for age, gender, BMI, phosphorus, Cr, PTH, hypertension, diabetes, smoking status and drinking status.

^b^ Linear analysis, *P*-value < 0.05 indicates a linear relationship

^c^ Non-linear analysis

^d^ *P*-value < 0.05 means Model B is significantly different from Model A, which indicates a non-linear relationship

Abbreviations: TyG, triglyceride-glucose; BMI, body mass index; Cr, creatinine; PTH, parathyroid hormone.
