# Supplementary material for: Albumin: a mediator of the association between serum calcium and triglyceride-glucose index among Chinese individuals with osteoporotic fractures
Source: Front Endocrinol (Lausanne). 2025 Sep 12;16:1574059. doi: 10.3389/fendo.2025.1574059 (PMC12463993; doi:10.3389/fendo.2025.1574059)
Supplement: Supplementary file 2 [file Table2.docx]

**Table S2.** Association between serum calcium and albumin in different models.

|  | Model ­1^a^  β (95% CI) *P*-value | Model ­2^b^  β (95% CI) *P*-value | Model 3^c^  β (95% CI) *P*-value |
| --- | --- | --- | --- |
| Albumin, g/L |  |  |  |
| Calcium, mmol/L | 17.041 (15.813, 18.269) <0.001 | 17.027 (15.796, 18.258) <0.001 | 17.168 (15.926, 18.410) <0.001 |
| Calcium 4 quantiles |  |  |  |
| Q1(1.47-2.12) | Reference | Reference | Reference |
| Q2(2.13-2.20) | 2.089 (1.611, 2.567) <0.001 | 2.088 (1.610, 2.566) <0.001 | 2.083 (1.607, 2.559) <0.001 |
| Q3(2.21-2.29) | 3.590 (3.112, 4.067) <0.001 | 3.588 (3.110, 4.065) <0.001 | 3.631 (3.151, 4.110) <0.001 |
| Q4(2.30-2.91) | 5.687 (5.210, 6.164) <0.001 | 5.681 (5.204, 6.159) <0.001 | 5.734 (5.252, 6.216) <0.001 |
| *P* for trend | <0.001 | <0.001 | <0.001 |

Association between serum calcium and albumin in different models. ^a^No adjustment. ^b^Adjusted for age, gender. ^c^Adjusted for age, gender, BMI, phosphorus, Cr, PTH, hypertension, diabetes, smoking status and drinking status. BMI, body mass index; Cr, creatinine; PTH, parathyroid hormone.
