# Supplementary material for: Albumin: a mediator of the association between serum calcium and triglyceride-glucose index among Chinese individuals with osteoporotic fractures
Source: Front Endocrinol (Lausanne). 2025 Sep 12;16:1574059. doi: 10.3389/fendo.2025.1574059 (PMC12463993; doi:10.3389/fendo.2025.1574059)
Supplement: Supplementary file 3 [file Table3.docx]

**Table S3.** Association between Albumin and TyG index in different models.

|  | Model ­1^a^  β (95% CI) *P*-value | Model ­2^b^  β (95% CI) *P*-value | Model 3^c^  β (95% CI) *P*-value |
| --- | --- | --- | --- |
| TyG index |  |  |  |
| Albumin, g/L | 0.023 (0.015, 0.030) <0.001 | 0.023 (0.015, 0.031) <0.001 | 0.025 (0.017, 0.033) <0.001 |

Association between Albumin and TyG index in different models. ^a^No adjustment. ^b^Adjusted for age, gender. ^c^Adjusted for age, gender, BMI, phosphorus, Cr, PTH, hypertension, diabetes, smoking status and drinking status. TyG, triglyceride-glucose; BMI, body mass index; Cr, creatinine; PTH, parathyroid hormone.
