# Supplementary material for: Albumin: a mediator of the association between serum calcium and triglyceride-glucose index among Chinese individuals with osteoporotic fractures
Source: Front Endocrinol (Lausanne). 2025 Sep 12;16:1574059. doi: 10.3389/fendo.2025.1574059 (PMC12463993; doi:10.3389/fendo.2025.1574059)
Supplement: Supplementary file 4 [file Table4.docx]

**Table S4.** Subgroup analyses exploring the association between serum calcium and TyG index.

| Subgroup | N | β (95%CI) | *P-*value |
| --- | --- | --- | --- |
| Gender, N (%) |  |  |  |
| Female | 1147 | 1.005 (0.728, 1.282) | <0.001 |
| Male | 393 | 0.734 (0.311, 1.156) | <0.001 |
| FLS |  |  |  |
| Yes | 530 | 0.923 (0.528, 1.318) | <0.001 |
| No | 1010 | 0.912 (0.624, 1.199) | <0.001 |

Note 1: Above model adjusted for age, BMI, phosphorus, Cr, PTH, hypertension, diabetes, smoking status and drinking status.

Note 2: In each case, the model is not adjusted for the stratification variable.

FLS, Fracture Liaison Service
